# Women’s Empowerment, Food Security, and Nutrition Transition in Africa

**DOI:** 10.3390/ijerph20010254

**Published:** 2022-12-24

**Authors:** Mosses Lufuke, Yunli Bai, Shenggen Fan, Xu Tian

**Affiliations:** 1 College of Economics and Management, Nanjing Agricultural University, Nanjing 210095, China; 2 Department of Economics, The University of Dodoma, Dodoma P.O. Box 259, Tanzania; 3 Key Laboratory of Ecosystem Network Observation and Modeling, Institute of Geographic Sciences and Natural Resources Research, Chinese Academy of Sciences, Beijing 100101, China; 4 United Nations Environment Programme-International Ecosystem Management Partnership (UNEP-IEMP), Beijing 100101, China; 5 College of Economics and Management & Academy of Global Food Economics and Policy, China Agricultural University, Beijing 100083, China

**Keywords:** women’s empowerment, food security, nutrition transition, Africa

## Abstract

Despite mounting recognition of the essential role of women’s empowerment in household dietary and nutrition changes, the diversity of culture across African countries presents ambiguity as to whether its impact is experienced homogeneously across the continent. This article presents a systematic review of whether women’s empowerment changes household dietary patterns, contributes to nutrition improvement, and consequently affects diet-related health outcomes in Africa. We find that whilst more research needs to be conducted, particularly with improved methodologies that can establish cause–effect relationships, there is consensus among the literature on the link between women’s empowerment and some domains of food security and dietary improvement. Meanwhile, studies on women’s empowerment and the additional demand pressure on some food categories are quite limited. This exacerbates the challenge of setting production plans that aim to address the continent’s question of food.

## 1. Introduction

In the past decade, the nourishment position in Africa has deteriorated remarkably, thus presenting the question of whether the region can achieve sustainable development goal (SDG) 2 of food security and nutrition. According to FAO [1], the percentage of undernourished people in the continent, with respect to the rest of the world, rose from 32.8% to 36.3% between 2014 and 2020. Meanwhile, as of 2020, the prevalence of severe food insecurity stands at 22.8% of the continent’s total population, which is more than twice the world average of 10.5%. Likewise, although lower than the world average, the incidence of obesity among adults in Africa has been substantially rising since 2000. By 2016, the percentage of the continent’s obese adult population was 12.8%, compared to 7.9% in 2000. The stunting among children under the age of five, although it has been decreasing, also remains far above the world average. It is estimated that, as of 2020, 30.7% of African children were stunted compared to 22% across the entire world.

Furthermore, the African continent is exemplified by numerous initiatives for women’s empowerment due to the presence of various layers of gender challenges (that is, gender inequality, inequity and other discriminatory norms), which are associated with a number of problems on the continent, including nutritional challenges. By empowerment, we imply attainment of the power for self-determination, affording the pursuit of a lifestyle based on one’s own value judgements. In order to achieve such power for self-determination, policymakers have worked in different domains, and consequently, scholars use these domains to assess the degree of empowerment in society. These domains of empowerment include production, resources, income, leadership and time. Women’s empowerment, as a result, involves influencing change in at least one of the highlighted domains for this gender group. More specifically, in this article, we define women’s empowerment as women’s gaining of control (not at the expense of men’s control) over any of the domains stated above. These initiatives and programs include, inter alia, Growth and Economic Opportunities for Women (GrOW), Affirmative Finance Action for Women in Africa (AFAWA), the Sahel Women’s Empowerment and Demographic Dividend project (SWEDD) and Africa Code Week’s Women Empowerment Program (ACW-WEP). 

The rationale behind women’s empowerment rests on the notion that when women attain the power of self-determination, social and economic improvements in various dimensions are more likely to appear, a concept that has been empirically substantiated [2,3,4]. Moreover, as women normally devote a large share of their incomes to their families [5,6,7], women’s empowerment, particularly in the economic domain, is considered as one of the channels for improving household living standards, which includes nutrition improvement. As a result, women’s economic empowerment has been a centerpiece of policy discourse in African countries. The continent is troubled by previously covered aspects of gender inequality and inequity ranging from discriminatory laws and norms to practices that persistently influence women’s outcomes [8]. For instance, in agriculture, the main economic sector in Africa, hidden aspects of gender inequality and inequity worsen women’s agricultural land ownership to just 12% despite occupying approximately half of the continental agricultural workforce. It was also reported that Africa has an approximately USD 42 billion financial gender gap, with 70% of all women being financially excluded [8]. These layers of challenges, consequently, raise the question of whether women’s empowerment on the continent can yield comparable results to other parts of the world. An additional motivation for this article on Africa rests in its higher degree of double nutrition burden compared to most other parts of the world. Statistics show that undernutrition and micronutrient deficiencies are not only the main concerns; rather, the state of overweight is also alarming, especially among adolescents and young women [9]. This—together with the data challenge, which affects the literature’s richness—rationalizes our motive to undertake this topic in Africa.

This article, consequently, provides a review of the nexus between women’s empowerment, household food security, the nutrition transition and diet-related health in Africa. We aim to understand whether women’s empowerment can address household dietary hurdles in Africa in a similar manner to the way it does in most parts of the world.

The next section presents the method that was used to select primary studies in this article. Section 3 examines empirical evidence on the relationships between women’s empowerment, various dimensions of household food security dynamics, and nutrition transition. Specifically, the section covers the nexus between women’s empowerment, on one side; and household food security, dietary patterns, dietary diversity, nutritional improvement—including household food allocation and diet-related health—on the other side. Section four illustrates the potential mechanisms that explain the association between women’s empowerment and the various dimensions of the household’s food subsector. The final section concludes this article.

## 2. Method

By mapping the existing evidence along the lines of women’s empowerment, food security and nutritional transition in Africa, we have made a comprehensive review of the literature published in scientific databases (SCOPUS, AgEcon Search, Asian development Review, RePec, EconLit., WorldCat), websites of institutions (IFPRI, World Bank, CGIAR) and references from the literature’s bibliographies. To ensure extensive searching, we selected a combination of search terms related to women’s empowerment, food security, dietary diversity, westernization of diet, nutrition improvement, household food allocation, diet-related health and Africa. The search was conducted from April to November 2022. The final search generated 201 original articles from the above-mentioned sources, 93 of which were considered potentially relevant, based on their titles. After reading the abstracts and subsequently the full texts, only 36 articles with full-text publications and coverage of Africa were discovered to be relevant. We then mapped the studies into six groups—household food security, westernization of diet, dietary diversity, nutritional improvement, household food allocation and diet-related health—in connection with women’s empowerment. Our mapping was simply based on whether a study agrees or disagrees about the existence of a relationship between women’s empowerment and the six dimensions of the food/nutrition dynamic. This makes our study read more like social science-type literature than a conventional systematic review. As indicated in Table 1, a total of 36 studies (excluding citations from other parts of this article) were considered relevant and were included in this review.

## 3. Women’s Empowerment, Household Food Security, and the Nutrition Transition

### 3.1. Women’s Empowerment and Household Food Security

Africa has twice the number of people who experience hunger than any other region in the world [10]. Improving food security in the region has therefore been at the center of both national and international efforts. Women’s empowerment is among the interventions that have been undertaken, with the view that a power balance in the household leads to a healthier allocation of resources and thus can mitigate a number of household setbacks, including food challenges. Empirical data from the continent mostly support the assertion that an improvement in food security is positively associated with the majority of the domains of women’s empowerment. Specifically, the literature [11,12,13] acknowledges the role of empowerment in terms of physical capital and economic agency, sociocultural factors such as education, and cultural values on the enhancement of food security within families. In South Africa, for instance, women’s empowerment in terms of the three mentioned areas has reduced the vulnerability of food insecurity in the form of household food consumption expenditure per adult [14]. Similarly, in Ghana, empowerment through education and decision-making was shown to enhance food security measured by the food insecurity experience scale (FIES) [15].

Surprisingly, despite the pronounced consensus on the positive associations between most of the indices of empowerment and food security, there is ambiguity on the efficacy of financial capital empowerment. In South Africa, for instance, women’s financial capital empowerment seemed to increase the possibility of households being food insecure in the future [14]. It has been argued that with high scales of financial capital empowerment, women tend to rarely invest in other capital assets, thus becoming prone to future food insecurity.

Generally, experiences from the region show that the severity of food insecurity can certainly be minimized through women’s empowerment, using different empowerment channels. We need to be mindful of the fact that most of the literature in Africa has focused on women’s empowerment in agriculture (WEIA). Therefore, more research is needed to provide extensive coverage of women across the economy. 

### 3.2. Women’s Empowerment and the Increasing Popularity of a “Western Diet”

Women’s increasing role in families is also associated with the dynamics of household food consumption [4,16,17]. It is argued that women possess a distinctive food taste and preference to men, largely due to their prominent kitchen role and, consequently, their vast knowledge of dietary issues [16,17]. Additionally, women’s empowerment by itself may facilitate fresh knowledge creation, specifically in terms of nutritional education, which in turn can augment the demand for certain foods. This is well-explained by Kadiyala et al. and Malapit et al. [18,19] in the fourth of six popularly recognized pathways between agriculture and nutrition. Therefore, while it is imperative to understand the role of women’s empowerment in household nutrition improvement, it is likewise important to understand whether such empowerment comes with consumption pressure for certain food items. 

One notable observation on food consumption changes, following women’s empowerment, is the rapid dietary change in low- and medium-income countries (LMICs) toward “Western diets” [20,21,22]. This means an increase in the intake of refined carbohydrates, fats, added sugar and animal-sourced foods at the expense of diets rich in coarse grains, vegetables and legumes. Largely, these westernized diets, which are attracting attention in LMICs, intensify the risk of certain health problems. Essentially, the food consumption convergence certainly deepens the paradox between women’s empowerment and dietary nutritional improvement in the society. Westernized foods such as bread, pasta, candy, cakes, cookies, ice cream, butter and others are increasingly becoming common in LMICs and attract women more than men [23,24]. The fact that production in LMICs is characterized by labor-intensive work means that men are still proportionally more loyal to traditional foods rich in starch than women [25]. Moreover, according to Kadiyala et al. [18], through pathway 2, income is an important determinant in influencing nutritional outcomes. Women’s limited income due to low participation in economic activities [8] might also explain the difference in the highlighted food consumption between gender. Both these conditions are fairly distinctive in LMICs, particularly in Sub-Saharan Africa (SSA) [26], making it difficult to form a conclusion on the association between women’s empowerment and dietary and nutritional improvements.

Several studies have quantified the differences in food consumption patterns between men and women in Africa. For instance, Ambikapathi et al. [27] revealed that the daily meal for men contained a higher proportion of energy than the meal for women. This difference in food preferences between gender is also observed in Berbesque [28]. In this study of a hunter–gatherer community, a preferential difference was observed for berries and meat, whereby women preferred the former more than the latter, while men preferred the opposite. Similarly, Plataroti [29] showed that women in Tanzania preferred to consume less sugar and fewer alcoholic drinks and more vegetables and fruits than men. Although these three studies do not discuss women’s empowerment, their findings certainly illustrate the likelihood of household dietary substitution when empowerment occurs. With women’s empowerment, there is the possibility for the household to switch consumption toward foods that are highly preferred by women.

Therefore, although studies that empirically analyze the cause—effect relationship between women’s empowerment and food consumption changes in African households are limited, the existing body of literature slightly indicates the possibility of switching diets following women’s empowerment. There is a need to carry out in-depth analysis in order to determine whether causality actually exists.

### 3.3. Women’s Empowerment and Dietary Diversity

A household’s dietary diversity is another area, apart from general food security, with which Africa contends. Inadequate dietary diversity has aggravated regional epidemiological transition. According to the *Global Nutrition Report* [30], an average of 9.7% and 9.9% of adult men and women, respectively, live with diabetes. Likewise, 9.2% and 20.7% of adult men and women, respectively, suffer from obesity, and the cases of mineral and vitamin micronutrient deficiency are still alarming. Furthermore, approximately 13.7% of all infants are born with a low weight.

Furthermore, given their prominent roles in household management, empowering women is equally intended to challenge the problem of limited diets in families. In Africa, experiences of women’s empowerment have been largely supportive, with only minor indecisive observations. In Nigeria, for instance, Olumakaiye and Ajayi [31] revealed that women’s empowerment through education increased household food diversity, which was measured using the food frequency table. Similarly in South Africa, using a list of 17 foods, Murugani and Thamaga-Chitja [14] disclosed that women’s input into production and public speaking increased dietary diversity. Moreover, in assessing the number of food groups consumed by households in a 24 h period, Quisumbing et al. [32] posited that empowerment in income decisions and autonomy in production was associated with household dietary diversity in some selected African countries. Chege et al. [33] posited that women’s empowerment in agriculture in countries of East Africa led to a more diverse diet in households. In Ethiopia too, Yimer and Tadesse [34] disclosed that all ten domains of WEIA resulted in households’ diversity of diets. Flodqvist [35] claimed that empowering women, particularly through nutritional education, led to more diverse and balanced diets in Tanzania. According to O’Neill [36], women generally invested larger proportion of their income in their families than men. Thus, with empowerment and increased control over resources, it is rational for the underlined literature to indicate the presence of a positive association between women’s empowerment and dietary diversity.

On the other hand, as with the case for food security, women’s empowerment in terms of access to credit has occasionally been witnessed to reduce dietary diversity, particularly in South Africa [14]. It is argued that women, especially in African rural areas, are more involved in informal money-lending schemes, as these areas have limited financial capacity to borrow large amounts of money [14]. As a result, the money borrowed is insufficient for procuring the inputs of production. They instead borrow as a coping strategy during food insecurity periods. 

### 3.4. Women’s Empowerment and Nutritional Improvement

Overall, there is a clear consensus among existing studies in Africa on the association between women’s empowerment in different contexts and nutritional improvements, be it at household level or for children or women themselves. Galie et al. [37], for instance, concluded that women’s control of resources in Tanzania generated a relatively higher proportional of household expenditure than men’s control of resources. Given the fact that women take better care of the household, it is implied that more control over resources for women may lead to an improvement in diet and nutrition. A similar qualitative study by Mindy et al. [38] ascertained that women perceived empowerment through control over income as the necessary pathway to improving family nutrition. Further research by Galie et al. [39] revealed that food security in terms of the household food insecurity access scale (HFIAS) and nutrition were positively associated with three domains (access to and control over land and livestock, control and use of income and workload, and control over own time) of empowerment. Additionally, Jones et al. [40], realizing that recommended body mass index (BMI) and blood hemoglobin level (Hb) were associated with healthy dietary intake, undertook a study in East African countries on their nexus with women’s empowerment. All three indices (instrumental agency, intrinsic agency and assets) of empowerment that were used in the study were positively associated with BMI and Hb, suggesting that empowerment may act as a crucial driver of nutritional improvement. Furthermore, underlying the importance of empowerment, a milk-based intervention research study [41] noted that unless women had control over resources, the intervention did not necessarily cause nutritional improvement, particularly among children. Other studies [42,43] also disclosed the existence of positive relationship between women’s empowerment and household nutritional improvement.

Despite the usefulness of these results, especially regarding their consistency, the qualitative nature as well as the light methods of correlation and regression analyses employed by the existing studies hindered the establishment of the empirical mechanisms that explained this nexus of women’s empowerment and the observed nutritional improvement. This lack of clear understanding of the mechanisms through which empowerment affects household dietary diversity and nutrition, as pointed to by Galie et al. [39], may weaken concerted efforts towards the realization of the fifth sustainable development goal. Therefore, while there is enough evidence on the relationship between women’s empowerment and household nutrition and dietary improvements, it is still worth conducting further research that will assess the impact of each channel, especially for policy reasons.

### 3.5. Women’s Empowerment and Household Food Expenditure Allocation

An additional and important observation in regard to the improvement of dietary diversity is the food consumption and expenditure allocation in households. The question of which specific food groups are becoming increasingly popular following women’s empowerment is vital. Given that the region is already in a dire situation of food insecurity, the increasing demand for some food groups might further escalate the problem. Similarly, since Africa is registering a mounting number of women’s empowerment programs, the knowledge of whether empowerment leads to a rising demand for certain food groups is crucial for firms in deciding what to produce.

The previous global literature posits that empowering women, especially through the economic domain, has led to improvements in both child and general household nutritional status as compared to when men are the sole income contributors [40,44,45,46,47]. One notable study about women’s empowerment and the affected food groups in terms of increasing demand was by Onah et al. [43], which included five Sub-Saharan African countries. The study employed the WEIA, with its ten domains. It established that empowerment in terms of autonomy for women who were cohabitating with their husbands increased the consumption of vitamin A-rich leafy greens, vitamin A-rich fruits and vegetables, dairy and dairy products, flesh protein, and eggs. On the other hand, empowerment in terms of the inputs of production increased the consumption of vitamin A-rich products, dairy and dairy products, fruits and vegetables, grains, and tubers. Lastly, empowerment in public speaking was associated with an increase in the consumption of fruits and vegetables and vitamin A-rich products. The demand magnitude of these food groups, however, varied from one country to another.

Despite the limitation of the studies on the variation in demand for particular food groups following women’s empowerment, an earlier-cited reference certainly illustrated the possibility of increased demand for some food groups. This implies that, with an increase in women’s empowerment initiatives, the demands for some food groups in Africa are estimated to further improve. Meanwhile, from the above-cited reference, women’s empowerment seems to strengthen the demand for healthy food groups.

### 3.6. Women’s Empowerment and Diet-Related Health

The quality of a diet plays an essential role in mitigating many chronic diseases, such as hypertension, diabetes, certain types of cancer, and cardiovascular diseases. Some human health disorders trace back to childhood feeding practices; thus, a proper diet and food consumption during infanthood is regarded as preventive therapy for future health problems. In this regard, the question of whether women’s empowerment can improve household health status through diet is of particular importance. Observations from empirical studies have indicated that women’s economic empowerment increases dietary diversity, as well as the World Health Organization (WHO)’s measurements of minimum acceptance diets and minimum meal frequency among household children in SSA [47]. Another study in Ghana revealed that empowering women through the ownership of assets, access to and decisions on credit, leisure time, autonomy in production, and group membership was associated with a healthy BMI score [48]. Similarly, Delano [12] argued that women’s empowerment through education positively affected yields, thus reducing malnutrition cases in Africa. Equally, Quisumbing et al. [32] posited that empowerment in terms of autonomy in production, decisions in agricultural activities, income decisions and ownership of agricultural assets were associated with lower BMI, while public speaking and leisure led to a higher BMI in Africa. The findings of these works reflected the observations in Section 3.2 that associated women’s empowerment with increased demand for healthy foods. Wu [48] found that children in families where the mother was the head of the household or households where the mother had higher bargaining power had higher BMIs than their counterparts whose mothers had less power.

Table 2 presents a summary of the six topics and findings for all 36 studies that were used in this article.

## 4. The pathways between Women’s Empowerment and Various Dimensions of Food Dynamics

The existing literature [18,19,49] recognizes six agricultural interventions that impact nutrition outcome. Three of them relate to women’s empowerment and can be applied to non-agricultural interventions as well. The literature, first, identifies household division of labor as an important pathway to influencing nutritional outcomes, and that women participate in more time-consuming activities, which have an adverse effect on nutrition-related cases. Second, the literature points to household allocation of resources as the other potential pathway. It is depicted that scant resource allocation towards women affects their access to means of production and, consequently, impairs household nutritional improvement. Last is decision-making power, which is highly determined by income, with the literature suggesting that income inequality and inequity between genders place women’s role in food and nutrition improvement at risk. Women’s empowerment, therefore, normally aims to work through these pathways.

**Figure 1:**
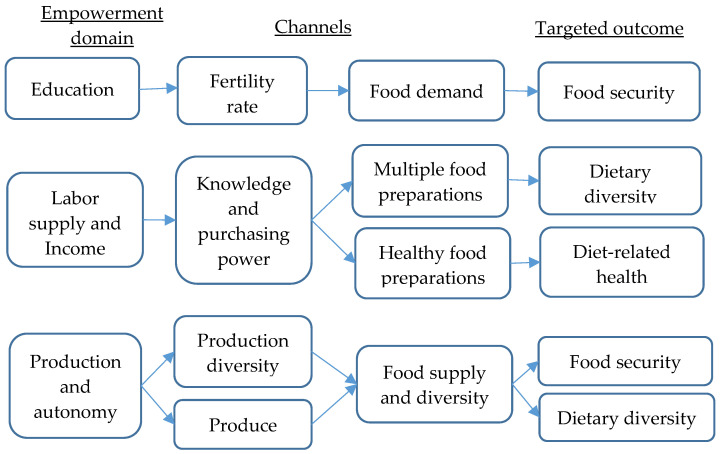
Channels for the impact of women’s empowerment on household food security and nutrition.

Figure 1 presents an intuitive way of showing the main channels, in line with pathways stated above, through which women’s empowerment may affect household food security, dietary diversity and diet-related health.

In food security, for instance, it has been reported that women’s empowerment in education reduces the fertility rate, in turn diminishing the overall food demand and easing regional food pressure [12,50]. Similarly, empowerment through inputs of production triggers yield abundance and a higher supply of foods that strengthen the status of food security [33]. 

As for dietary diversity, Chege et al. [33] disclosed that empowerment in terms of the autonomy of production led to dietary diversity through the expansion of production diversity. This indirect link between autonomy in terms of production and household dietary diversity, however, requires further analysis. This is because experience shows that, in Africa, higher agricultural production does not necessarily lead to an improved household diet. In Tanzania, for instance, statistics have shown that regions considered the country’s food baskets are the ones with the highest prevalence of child stunting [51,52]. Furthermore, the mechanisms for dietary diversity rest on the fact that, with education, women can attain skills in the use of technologies, which eases the cooking process, thus encouraging the preparation of multiple foods.

The prominent mechanism of the nexus between women’s empowerment and diet-related health is underpinned by the concept of healthy food consumption. That is, with education and income empowerment, women gain knowledge on healthy food preparation and consumption. This, consequently, reduces the risk of contracting diet-related diseases among household members. This is why Onah et al. [46] acknowledged an increase in the consumption of healthy food categories when women are empowered. However, further research would be useful, especially research that indicates the sources of food categories among households. This is because documenting the increase in consumption of certain food categories might likewise be contributed to by increased accessibility rather than empowerment.

## 5. Conclusions

With their prominent role in the kitchen and household food preparation, women offer an important avenue for addressing several challenges in relation to the food subsector. Empowering them with the necessary capabilities can lead to changes in various aspects of households’ dietary patterns. Studies of Africa indicate that empowering women through physical capital, economic agency, and sociocultural factors can have a positive influence on either food security, diet improvements or the enrichment of diet-related health. Some domains of women’s empowerment also exert household demand pressure on some food categories more than others. Notably, because studies show that empowerment in different domains has different outcomes, empowerment programs have to be specific in terms of their forms and targeted results.

It is evident that studies on the relationship between women’s empowerment and food security, as well as the nutrition transition in Africa, are limited. Moreover, most studies have mainly been qualitative in nature, thus failing to establish causation. Moreover, these qualitative studies are also insufficient for generating population-level interpretations. The existing analytical studies, on the other hand, have largely employed correlation or ordinary regression analyses, which do not imply cause and effect relationships between women’s empowerment and dietary and nutritional improvements. For this reason, it is evident that well-designed, prospective further studies are needed, ideally within different empowerment domains and causation outcomes. Furthermore, as depicted before, women tend to have particular preferences for the consumption of certain food items compared to men. In addition to the lack of research on the relationship between empowerment and household consumption of specific food items, the existing studies also do not account for the impact of confounders. One notable confounder is the uniqueness of women’s preferences for some food items. Therefore, a need for further research that can evaluate whether women’s empowerment supplements already existing preferences is important. Finally, empirical studies in Africa are quite homogeneous, making it difficult to determine potential results if the studies were conducted in different settings. For example, most studies have adopted the WEIA approach, excluding non-agricultural women as a result. Meanwhile, apart from FIES, which has also been used sparingly, empirical studies on the applications of other experience-based food insecurity scales are not common in the region. For informed decision-making vigor, more research needs to be carried out, as noted in the sections above.

## Figures and Tables

**Table 1:** Studies included in this review.

Topic of Coverage	Number of Studies
Women’s empowerment and household food security	9
Women’s empowerment and increasing popularity of Western food	4
Women’s empowerment and dietary diversity	5
Women’s empowerment and nutrition improvement	9
Women’s empowerment and household food allocation	3
Women’s empowerment and diet-related health	6
Total	36

**Table 2:** Main findings of primary studies that were reviewed.

Author(s)	Topic	Summary of Findings
Sharaunga et al. [11]; Delano [12]; Essilfie et al. [13]; Murugani and Thamaga-Chitja [14]; Ross et al. [15]	Women’s empowerment and food security	Positive association, except for capital empowerment
Delgado et al. [23]; Ambikapathi et al. [27]; Berbesque [28]; Plataroti [29]	Women’s empowerment and Westernized diets	Less energy rich food, light food, vegetable and fruits
Murugani and Thamaga-Chitja [14]; Olumakaiye and Ajayi [31]; Quisumbing et al. [32]; Chege et al. [33]; Flodqvist [35]	Women’s empowerment and dietary diversity	Positive association, except for empowerment in access to credit
Ambikapathi et al. [27]; Quisumbing et al. [32]; O’Neill [36]; Galie et al. [37]; Mindy et al. [38]; Galie et al. [39]; Jones et al. [40]; Lentz et al. [42]; Onah et al. [43]	Women’s empowerment and nutrition improvement	Positive association, but women need to have control over resources
Opata et al. [6]; Jones et al. [40]; Onah et al. [43]	Women’s empowerment and household food expenditure allocation	Additional expenditure on vitamin A-rich food, vegetables, dairy products, flesh proteins and eggs
Giancola et al. [9]; Delano [12]; Ross et al. [15] Quisumbing et al. [32]; Onah et al. [43]; Na et al. [47]	Women’s empowerment and diet-related health	Positive association for large number of empowerment domains

## Data Availability

Not applicable.
